# OTX2 regulates the expression of TAp63 leading to macular and cochlear neuroepithelium development

**DOI:** 10.18632/aging.100839

**Published:** 2015-11-10

**Authors:** Ramona Palombo, Giovanni Porta, Ernesto Bruno, Paolo Provero, Valeria Serra, Karthik Neduri, Andrea Viziano, Marco Alessandrini, Alessandro Micarelli, Fabrizio Ottaviani, Gerry Melino, Alessandro Terrinoni

**Affiliations:** ^1^ Department of Experimental Medicine and Surgery, University of Rome "Tor Vergata", 00133 Rome, Italy; ^2^ Department of Experimental and Clinical Medicine, Università dell'Insubria, via Dunant 5, 21100 Varese, Italy; ^3^ Department of Clinical Sciences and Translational Medicine, University of Rome “Tor Vergata,” 00133 Rome, Italy; ^4^ Medical Research Council, Toxicology Unit, Hodgkin Building, Leicester University, Lancaster Road, P.O. Box 138, Leicester LE1 9HN, UK; ^5^ Biochemistry Laboratory, IDI-IRCCS-FLMM, c/o Department of Experimental Medicine and Surgery, University of Rome "Tor Vergata", 00133 Rome, Italy; ^6^ Department of Molecular Biotechnology and Health Sciences, University of Turin, 10126 Turin, Italy

**Keywords:** Ectodermal Dysplasia, TAp63, Cochlea, Macula, OTX2

## Abstract

OTX proteins, homologs of the *Drosophila orthodenticle (Otd)*, are important for the morphogenesis of the neuroectoderm, and for the central nervous system formation. OTX1 and OTX2 are important for the cochlea and macula development, indeed when OTX1 is knocked down, these organs undergo developmental failure. Moreover OTX2 transfection revert this effect in OTX1^−/−^ mice. The TA isoform of TP63, involved in Notch regulation pathway, has a critical function in the cochlear neuroepithelium differentiation. TAp63 positively regulates Hes5 and Atoh1 transcription. This pathway has been also demonstrated in p63^−/−^ mice, and in patients p63 mutated, affected by Ectodermal Dysplasia (ED, OMIM 129810). These patients are affected by mild sensorineural deafness, most likely related to the mutation in p63 gene impairing the Notch pathway. We demonstrated the role of OTX2 on TAp63 regulation necessary for the correct formation of macular neuroepithelium and we confirmed the impairment of vestibular function caused by p63 mutations. Although the abnormalities found in our patient were still at a subclinical extent, aging could exacerbate this impairment and cause a decrease in quality of life.

## INTRODUCTION

The OTX proteins, the vertebrate homologous of the *Drosophila* gene *orthodenticle (Otd)*, include an important class of Homeodomain-containing transcription factors which play an essential role in embryonic morphogenesis. Their functions are critical in specifying cell identity, in cell differentiation, and in the positioning of the bodily axis during embryo development [1]. They are involved in the induction and in the morphogenesis of the neuroectoderm, leading to the formation of the vertebrate central nervous system [2]. In particular, *OTX2* is involved in rostral head development, including olfactory, auditory and visual systems, [1], and it plays an important role in the specification and maintenance of the rostral neuroectoderm [3, 4]. The vertebrate inner ear originates from the otic vesicle, an invagination of the otic placode, from embryonic day 8 (E8) in mice, giving rise to sensory and supporting cells in both the cochlea and the vestibule. It is known that cellular pathways such as FGF, Notch and Wnt have been related to the formation of the otic placode and the differentiation into prosensory or non-sensory cellular lineage [5]. OTX1 and OTX2 have been described as genes important in the development of cochlea and macula [6]. These organs undergoes to abnormal developmental failure when OTX1 is knocked down. Moreover, the use of human OTX2 cDNA was able to revert these effects in OTX1^−/−^ mice, leading to think that it is the main controller. OTX2 is regulated during cochlea and vestibular apparatus by Gbx2 and Pax2 and the lacking of this control leads to cochlear and vestibular abnormalities [7, 8]. Otx2 gene is a crucial target of N-myc during inner ear development. Otx2 expression is lost in N-myc mouse mutants, and N-myc misexpression in the chick inner ear leads to ectopic expression of Otx2 [9].

Otic prosensory cells differentiate while acquiring a spatial disposition typical of the adult inner ear, with a basal layer of Sox-2 positive supporting cells underlying MyoVIIa-positive hair cells. While the expression of the transcription factor Atoh1 and Hes5 has been proven to be mandatory for the differentiation into hair cells. The vestibular maculae are specialized sensory epithelia that provide information about linear accelerations while sensing the head positioning in reference to gravity. Hair cells in the macular receptors, the utricle and the saccule, are divided by a central region named striola, in two groups with opposite stereociliary bundle orientations [10].

In some vertebrates and in experimental conditions, some macular hair cells are produced after birth, apparently due to a transdifferentiation from supporting cells, which seems to be promoted by inhibition of Notch signaling activity and subsequent regulation of Atoh1 [11] and Hes5 [12]. The role of Notch inhibition in activating the differentiation of new hair cells in humans is yet to be understood [13, 14].

The spatial distribution of sensory hair cell is fundamental in the macular receptors, whose correct functioning as gravisensors strictly depends on the correct orientation of the cilia in space; this process may be disrupted in several stages of the morphogenetic pathway [15].

We recently demonstrated that the differentiation of cochlear neuroepithelium is regulated by the TA isoform of TP63, which is involved via the regulation of Notch pathway. This regulation is achieved by TAp63, that regulates the transcription of Hes5 and Atoh1 [16, 17]. In mice lacking of TAp63, the Corti organ displays abnormalities respect the number of inner and outer hair cells (IHC, OHC). Specifically, the defect is represented by the presence of supernumerary IHC and OHC cells. Hearing tests of these mice showed that they are affected by moderate sensorineural deafness [17]. The pathway has been also investigated “*in vivo”* in Ectodermal Dysplasia (ED, OMIM 129810) patients, characterized by autosomal dominant mutation in p63 genes [18, 19]. About 17% of them showed also conductive deafness, but it has been demonstrated that part of patients with p63 mutations are also affected by mild sensorineural deafness, most likely related to the impairment of the Notch pathway due to the loss of activity of mutant p63 [17].

In the present study we analyzed “*in vitro”*, the role of OTX2 in regulating the expression of TAp63 transcription factor. To refine the OTX2 pathway also in relationship with vestibular organogenesis, we analyzed the development of this organ in p63^−/−^ mice using confocal microscopy analysis of the organ, and in a human ED patient, in which vestibular examination were performed.

## RESULTS

### “In silico” analysis of OTX responsive elements in the p63 gene

**Figure 1:**
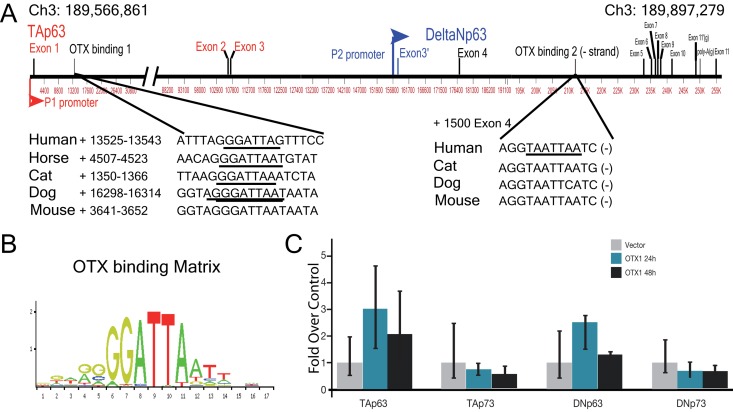
Promoter analysis of p63 gene (**A**) Genomic location and structure of human p63 gene, showing the position and size of the different intron and exons. In lower panels are identified, the location position of OTX responsive elements, and their conservation in different species. (**B**) Detailed structure of p63 gene. (**C**) OTX2 binding element, matrix Jaspar PH0130.1.

With respect to the existing data, which demonstrates the involvement of the TAp63 transcription factor in the organogenesis of the Corti organ [17], and the importance of OTX homeobox protein in the neural and sensory organs development, we analyzed the possibility of a relationship between the two transcription factors. In particular, since OTX1-2 are expressed in the early stages of embryogenesis, a downstream control of TAp63 by one of these transcription factors has been hypothesized. This analysis was first conducted *“in silico”*, searching for OTX responsive elements, using JASPAR matrix id PH0129.1 and PH0130.1, relative score > 0.9, represented by the motif illustrated in Fig 1B.

We found two of these sequences in the gene codifying for p63, laying in Ch3: 189,566,861-189,897,279. The first putative binding sequence is located inside intron 1, (+) strand, while the second is located within the intron 5, (−) strand (Fig. 1A). A conservation analysis was performed aligning genomic p63 sequences for different organism using the USC genome browser (Fig. 1A). The element in intron 5 showed a good conservation among species (Fig. 1A, lower right panel), while that in intron 1 did not show the same conservation. Indeed, the non-coding sequence of p63 intron 1 is bigger than 100Kb, and presumably it had more possibility of divergence during evolution in species. For this reason we performed an analysis using Jaspar website, looking for OTX binding sites inside intron 1 of different species. As result we identified an OTX responsive element present in the sequence of different mammalian organism, from human to mouse even if in a different position, meaning that also the element in (+) strand of intron 1 is functionally conserved.

### Molecular study of OTX2 responsive elements in the p63 gene

**Figure 2:**
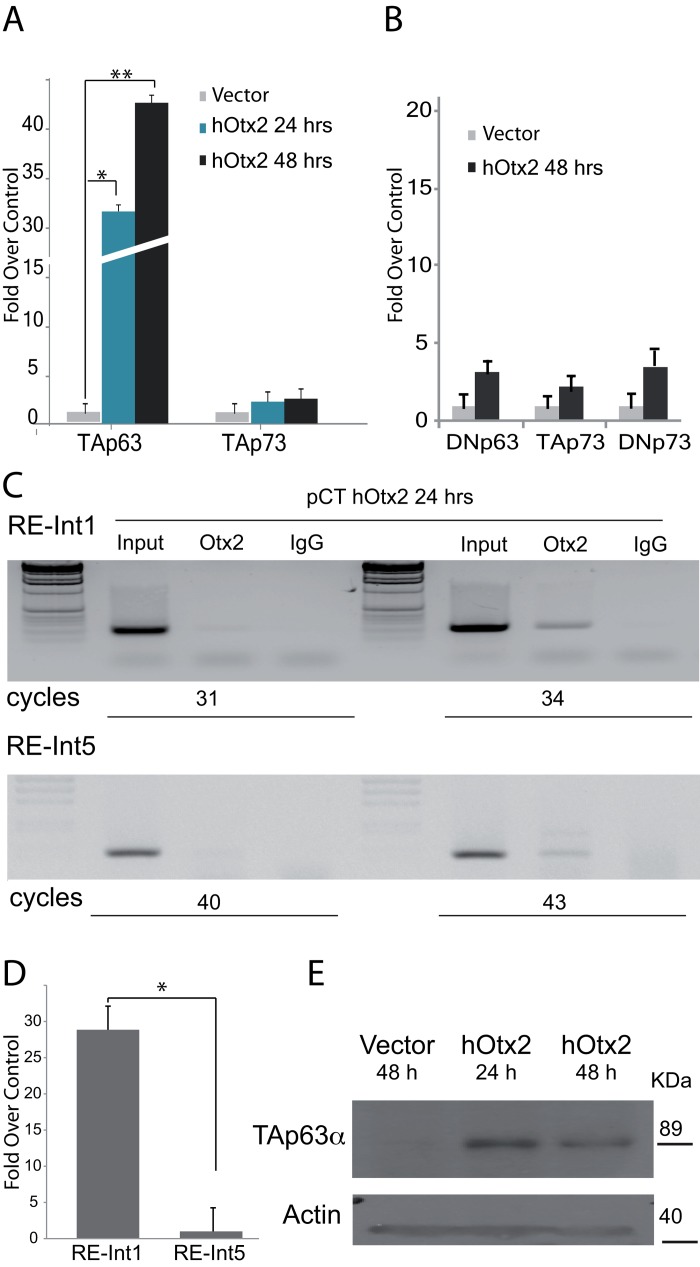
Molecular study of OTX2 responsive elements in the p63 gene (**A**) The H1299 cell line has been transfected using OTX2 cDNA, and the expression of p63, p53 and p73 analyzed at 24 and 48 hours by RTqPRC analysis. The graphs show that OTX2 nicely induces the transcription of TAp63 isoform, with very low effects on p53 and none on TAp73, *t-test on DDCt values, coupled, two tails, *p = 0,0005, **p = 0,00073. (**B**) Induction control respect to DeltaN isoforms, the graph show that 48 hours and none of these isoform are transactivated. (**C**) ChIP analysis, showing the binding of OTX2 at the responsive element, located at the 5′ (upper panel), respect to that located within intron 5. In the first case the PCR product is detectable at cycle 31 whereas the other is only detectable later after 43 cycles. **(D)** qPCR analysis using DNA pulled down using OTX antibody. *t*-test on DDCt values, coupled, two tails, *p = 0,00057.( E) Western blot of protein extracted from cells after the transfection. A band corresponding to TAp63 molecular weight is visible. The control has been obtained using protein extracts from a SaOS TET-On cell line.

Since OTX1 and OTX2 bind to a very similar consensus sequence, we first tested the ability of OTX1 in transactivating both isoform of p63 (Fig. 1C). As control we also tested its activity on the other member of the family p73, known to be involved in neural tissue differentiation [20]. OTX1 coding region in pCMV6 vector, has been used to test the transactivation ability in H1299 cells. The results show that OTX1 transcription factor is not able to drive the expression of none of the isoform of p63 and p73 (Fig. 1C). Furthermore we tested the transcriptional activity of OTX2 in driving p63 expression. OTX2 coding region [3] was used for transfection, then we analyzed the expression of p53 family genes after 24 and 48 hours. The results showed that OTX2 is able to strongly transactivate TAp63 and to a lower extent TAp73, indicating TAp63 as its possible transcriptional target (Fig. 2A). The analysis of DeltaN isoforms for both p63 and p73 showed a no significant increase at transcriptional control (Fig. 2B).

To understand which one of the two putative responsive elements found in the “*in-silico”* analysis is physically bound by OTX2, driving the expression of TAp63, we performed a ChIP analysis using chromatin of transfected cells pulled down using OTX antibody. The results showed that OTX2 principally binds with the responsive element in Intron 1 of the p63 gene (Fig. 2B, upper panel). In fact, a clear signal is visible at 34 PCR cycle on the other hand the band from the responsive element in Intron 4 is only visible over 43 cycles (Fig. 2C, lower panel). Furthermore, a qPCR performed for the two putative sites demonstrated that the element in intron 1 is present in the recovered chromatin in higher concentration respect to that in intron 5 (Fig. 2D), approx. 28 fold over the control.

Moreover the western blot analysis demonstrated that OTX2 is able to induce the expression at protein level of the TAp63 isoform only (Fig 2E), thus confirming that the responsive element located in intron 1 contributes actively to the transcription driven by the 5′ promoter, specific for the TA isoform.

### Involvement of OTX2 in vestibular macula organogenesis

The data obtained showing the control exerted by OTX2 transcription factor in TAp63 expression, demonstrates that OTX2 is upstream in control cascade respect to TAp63. This adds new information to the previously described mechanism by which TAp63 regulates the differentiation of sensorineural epithelium trough with the control of Hes5 and Atoh1 (Math1) [17], genes belonging to the Notch pathway.

**Figure 3:**
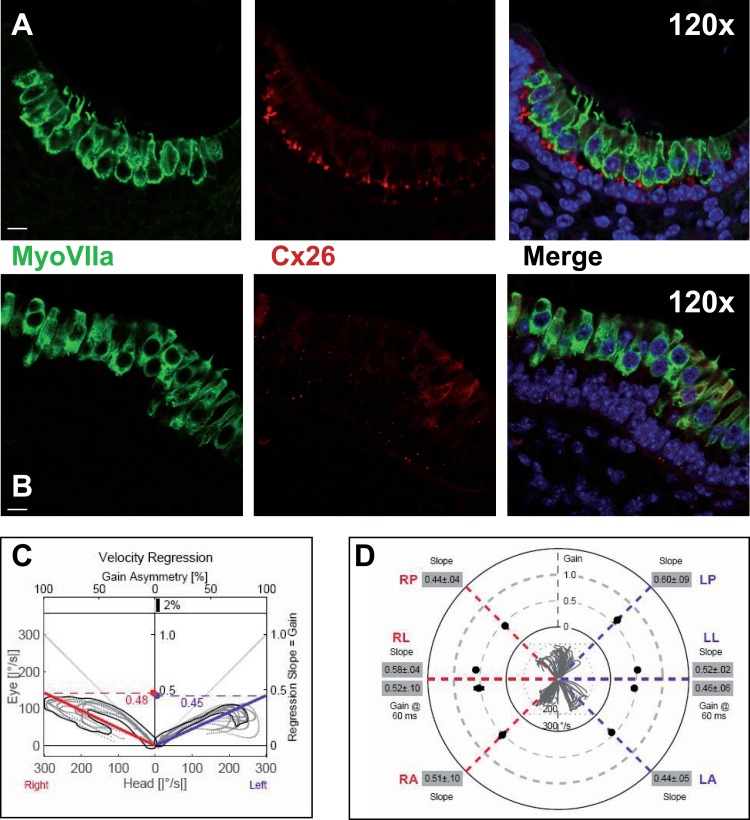
Macular structure in p63 defective mice and vestibular function in EEC patient (**A**) Representative picture of a macula of the vestibule from mice normal controls stained with MyoVIIa and Connexin 26 antibody. Dapi has been used for nuclei staining. Bars =20 μm. (**B**) Macular neuroepithelium from p63−/− mice. (**C**) Results of vHIT testing of the lateral semicircular canal (LSC) for the patient described in the article; the regression analysis of the vestibulo-ocular reflex (VOR) gain (eye angular velocity/head angular velocity) shows values of 0.48 and 0.45 for the right and left LSC, respectively. This shows VOR hyporeflectivity (average gain between 0.8 and 1.1). (**D**) The vestibular hyporeflectivity for both sides was confirmed by studying VOR gain for all the semicircular canals, with instant gain at 60 milliseconds for the lateral semicircular canals: gain values were all under average, without any significant asymmetry involving one side with respect to the other. Canals positioned on the same plane are connected by lines; RA= right anterior, RP= right posterior, RL=right lateral; LA= left anterior, LP= left posterior, LL=left lateral.

Since OTX2 pathway is also involved in the differentiation of the vestibular apparatus, we tried to verify that if in p63 ^−/−^ mice the morphology of the macula could be altered. Sections from p63 ^−/−^ and control mice were stained and analyzed in immunofluorescence by confocal microscopy, using MyoVII and connexin 26 antibodies. In the staining, ciliate and supporting cells are identified, including also the cells of basilar membrane, that is in direct contact with the ciliate cells. The results show a wide destructuration of the macular general architecture. In normal control, the cells positive to MyoVIIa are organized like in a pseudo-stratified epithelia, with the nuclei placed in the same row, and the stereocilia are clearly visible (Fig. 3A). The CX26 antibody nicely stains cells of the basilar membrane. In p63^−/−^ mice the macular neuroepithelium seems to be disorganized and detached from the basilar membrane (Fig. 3B), whereas the stereocilia are only partially present. Furthermore, CX26 expression is significantly impaired, as it has been demonstrated in cochlea *stria vascularis* [17].

These complex modification leads to hypothesize physiological problems in the organ when the OTX2/p63/Hes5-Atoh1 expression is impaired like in p63^−/−^ mice, or due to the presence of p63 mutations as in EEC patients.

### Clinical vestibular examination and physiology

To address this point, we performed otoneurological testing in a patient affected by ED. The subject, a 18-year old male, carrying the heterozygous R304W mutation in p63 gene, previously demonstrated to abolish its transactivating activity [21, 22]. The patient had a childhood history - at the age of 7 - of hearing loss, with previous middle ear ventilation tube positioning and no personal history of vertigo or unbalance.

The patient's otoscopy was unremarkable; impedance audiometry showed type A tympanogram (suggesting no significant pressure drop in the tympanic cavity) in both ears. The cocleo-stapedial reflex was present bilaterally for both ipsilateral and contralateral stimulations, at a threshold of 80 dB. Pure tone audiometry showed a mild conductive hearing loss in both ears at 0.25 and 0.5 kHz (maximum air-bone gap 15 dB, bilaterally, with a 5 dB bone conduction threshold) and a 25 dB air conduction threshold at 6 and 8 kHz for the left ear, while the right ear had a 15dBthreshold for both frequencies. VNG did not show any spontaneous or positional nystagmus and the head shaking test was negative. Caloric testing results, calculated by means of formula of Jongkees, were not significant for asymmetry, with values of 8% right prevalence and 4,5% right preponderance. However, the mean maximum SPV was 6°/s (degrees per second) for the right ear and 7°/s for the left ear warm stimulation and 4°/s and 3.5°/s for the cool stimulation, respectively. This values – below of standard scores – could indicate vestibular hyporeflexia, in absence of significant asymmetry [23]. The c-VEMPS analysis showed no evident P1 and N1 wave response bilaterally, even with repeated testing and after increasing the stimulus intensity up to 120 dB nHL. Regression analysis on vestibulo-ocular reflex gain, calculated by vHIT measures, demonstrated to be coherent with caloric test findings: no significant difference between the left and right labyrinth was found, both in the horizontal and in the vertical semicircular canals (Fig. 3C,D), with a mean VOR gain constantly below values usually observed in healthy controls (average gain between 0.8 and 1.1) [24].

## DISCUSSION

Development of sensorineural organs begins early during embryogenesis, and the homeobox gene OTX2 has a fundamental role in the development and differentiation of cochlear and vestibular apparatus. The Notch pathway is also involved, and in a previous work we demonstrated that TAp63 regulates some genes of this pathway (Hes5, Atoh1) in the development of cochlea [17].

**Figure 4:**
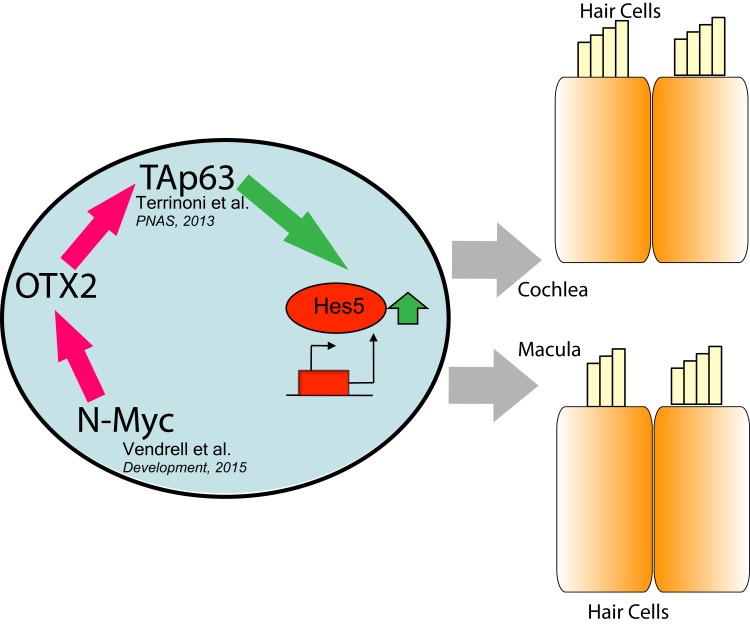
Schematic representation of OTX2 pathway N-Myc has been demonstrated to drive the expression of OTX2, and the latter is able to transactivate TAp63, inducing Hes5 and leading to the differentiation of both cochlear and macular neuroepithelium.

In this study we investigated the interaction between OTX2 and p63 transcription factor, in compelling also the correct vestibular organogenesis, adding some major information about the structure of the pathway. We demonstrated the involvement of OTX2 in the regulation of TAp63 expression, that places this transcription factor at the top of the regulatory cascade, that can be now identified as OTX2- > TAp63- > Hes5/Atoh1 (Fig. 4). In fact, the responsive element of OTX2 found in the 5′ regulatory region of p63, specifically regulates the TA isoform of the gene, and it is conserved among species. In p63^−/−^ animal model, in which the pathway is strongly compromised, the defects are evident at structural level. In mice lacking OTX2 expression, a similar defect in the organization of hair cells in cochlea has been demonstrated, with the appearance also of an ectopic sensory epithelium with supernumerary and disorganized hair cells [9]. Furthermore evident defects are present in the macula and saccule of mice OTX1^−/^−, and worsened in OTX2^−^/+ OTX1^−^/− [6]. The defect in OTX1^−^/− can be rescued overexpressing OTX2 cDNA, demonstrating a fundamental role of this gene in driving the correct architecture of the organ. In human patients carrying p63 mutations, probably there are not strong structural changes, because part of the functionality of the transcription factor is retained. However patients display impairment in sound transduction capabilities, and also how we demonstrated now, in vestibular functionality. In fact, to address this, we performed a functional evaluation of the vestibular organs, which has not yet been studied in subjects affected by ED. The clinical evaluation of a young ED patient with no particular complaints about hearing loss or vestibular symptoms showed subclinical impairment of inner ear function. We show, for the first time, in an ED patient who has no personal history of vertigo or dizziness, that the ampullary function is impaired at a subclinical extent. Moreover, the bilateral absence of recognizable P1 and N1 waves could suggest an impairment of the saccular function, involved in the vestibulo-collic reflex. This is coherent with previous findings about the role of Notch pathway in the vestibular receptors, the cristae and the maculae; congenital alterations in this signaling sequence could disrupt the complex architecture of these receptors, increasing the risk for vestibular impairment [11, 14].

Furthermore, this phenotype could represent a predisposition to severe decline in hearing and balance due to aging, given the physiological loss of hair cells that occurs over the years. Hair cell loss may be due to several causes, most frequently oxidative stress, noise exposure and ototoxic drugs; all of these mechanisms are amplified with aging.

To the best of our knowledge, this is the first attempt of an “*in vivo*” evaluation about connections between described morphological abnormalities and clinical data.

Thus, we suggest that more subjects affected by ED would undergo a vestibular examination analysing both the macular and the ampullary functions in order to have further insight regarding the extent of inner ear abnormalities related to p63 mutations. It may be of great help to assess these abnormalities before physiological hair cell loss due to aging. This condition can worsen the cochlear and vestibular function; thus, ED patients could receive in time the appropriate treatments to preserve hearing and balance.

## METHODS

All clinical investigation must have been conducted according to the principles expressed in the Declaration of Helsinki.

### “In silico” analysis

The Jaspar database has been used to determine the presence of OTX binding sites using matrix id PH0129.1 and PH0130.1

### Mice

p63 knockout mice are described in [18] and section are obtained as described in [17].

### Cell culture, transfection and plasmid

H1299 and SaOs-2 cells have been transfected using OTX2cDNA cloned in PCT-vector, using as control SaOs-2 TetON-TAp63alpha as described in [25, 26].

### Immunostaining and confocal microscopy

See [27, 28] for embryo preparation and confocal analysis. Subsequently, sections were incubated for 2 hrs with the following primary antibodies: anti-p63 (H129 sc-8344 and H137 sc-8243, Santa Cruz; 1/100 dilution), anti-MyoVIIa (25-6790, Proteus; 1/100 dilution), and anti-Cx26 (13-8100, Invitrogen; 1/100 dilution) see [17].

### Real Time qPCR, Semiquantitative RT-PCR and ChIP

See for PCR analysis and Supplementary table 1 for specific PCR primer sequences. H1299 transfected with OTX2 cells (5*10^6^) were crosslinked for 10 min, in a solution containing 1% formaldehyde and ChIP assays were performed using a MAGnify ChIP system (Invitrogen), and Immunoblot analysis were performed as described previously [17, 28].

Real Time as been performed using ABII 7500 instrument, using GoTaq^®^ qPCR Master Mix (Promega) and GoScript™ Reverse Transcription System (Promega) for reverse transcription. Primers Real Time: TAp63 (+)GGA CTG TAT CCG CAT GCA G, (−)GAG CTG GGC TGT GCG TAG; DNp63 (+)GAA GAA AGG ACA GCA GCA TTG AT, (−) GGG ACT GGT GGA CGA GGA G; TAp73 (+)CCT GGA GGG CAT GAC TAC ATC, (−)TTG TGC GTA GGG CGA GTG DNp73 (+)GGA GAT GGG AAA AGC GAA A, (−)GGA ACT GGT GTC CCG TGG. Primers ChIP: OTX2(p63int1) (+)ACA GAG CAA CAG AGG CAC TG (−) CAT GGA ATA ATT GAA GTC CCT TC; OTX2(p63int5) (+)CTG CTT GAT GTC AAC ATT TGT GGG, (−)GCA CTT TTC CTG ATG GCT CCA G.

### Western blot analysis

SaOs-2 cells line with doxycycline-inducible expression of HA-TAp63 has been used as TAp63 positive control. H1299 has been used for transfection of OTX1, and OTX2, and anti p63 antibody ((D2K8X) XP^®^ - Cell signaling 1/1000 dilution) has been used for WB.

### Patient

In a sound-proof room we performed pure tone audiometry (GSI 61 Clinical Audiometer, Grason-Stadler, United States) by calculating the hearing threshold for both ears in the frequency range from 0.25 to 8 kHz with both bone conduction and air conduction measures as well as impedance audiometry (GSI Tympstar, Grason-Stadler, United States). The vestibular clinical examination (spontaneous nystagmus, positional maneuvers, head shaking test) was performed by means of quantitative VNG with a camera connected to a software analyzing the data (EDM, Euroclinic, Italy). As regards caloric testing, asymmetry was calculated by the formula of Jongkees from the slow-phase velocity (SPV) and, according to Honrubia [29], vestibular paresis was defined as more than 25% asymmetry between the right-sided and the left-sided responses [30]. c-VEMPS were obtained by using the ICS Chartr EP 200 system (GN Otometrics, Denmark) registering P1 and N1 wave amplitude and ratio from an electrode placed on the medial aspect of the sternocleidomastoid muscle, in response to acoustic stimulation (rarefaction clicks at an intensity of 95 dB nHL, at a rate of 5.1 stimuli per second, for 60 seconds, repeated three times for each ear). VHIT responses were obtained by means of the EyeSeeCam system (EyeSeeCam^®^, Munich, Germany) with a camera positioned on special glasses secured to the patient's head, connected to a video-oculography software. The patient was instructed to stare at a visual target while impulsive head movement were administered, in the plane of both the horizontal and the vertical semicircular canals. The gain of the vestibulo-ocular reflex (eye angular velocity/head angular velocity) was calculated for both ears in each plane, and regression analysis was used to determine if any significant asymmetry between the vestibular endorgans was present.
